# Temporomandibular joint involvement (TMJ), a silent disease with severe alterations in young adulthood patients affected by juvenile idiophatic arthritis (JIA)

**DOI:** 10.1186/1546-0096-9-S1-P174

**Published:** 2011-09-14

**Authors:** Falcini Fernanda, Melchiorre Daniela, Ceri Lorenzo, Capannini Serena, Denaro Valentina, Biondi Katia, Bosco Mario, Matucci Cerinic Marco

**Affiliations:** 1 Department of Internal Medicine, Section of Rheumatology, Transition Clinic, University of Florence, Florence, Italy; 2 Department of Odontostomatologic Sciences, University of Pavia, Pavia, Italy

## Background

TMJ involvement has been largely reported in all subsets of JIA. At onset, it may be subtle and asymptomatic leading, when unrecognized and untreated, to severe joint alterations in early adulthood. The reported prevalence of detectable radiographic changes of TMJs varies from about 30% to 63% and 50-80% of children with JIA will have evidence of TMJ arthritis by MRI and by sonographic exam (SE) (effusions, synovial enhancement, condylar flattening and/or erosions) before evidence of X-ray damage. Pts with oligoarticular JIA onset (O-JIA) seem to be at higher risk of developing TMJ damage and, at young adulthood, when peripheral arthritis may be quiescent, suddenly complain of TMJ disease symptoms.

## Aim

To evaluate facial asymmetry and TMJ involvement in a cohort of young adults with JIA.

## Methods

Our study population included 80 consecutive pts (58 F, 22 M, mean age 14,5+/-4,4 yrs), mean age at JIA onset 6,9+/-5 yrs, mean disease duration at first orthodontic evaluation 7,7+/-5,2 yrs, fullfilling the ILAR criteria for JIA, all treated at Transition Clinic of Rheumatology Department between June 2008 and December 2010. Out of 80 pts, 40 had O-JIA, 27 polyarticular (P-JIA), 4 systemic (S-JIA), 9 enthesitis-related (ERA-JIA) onsets. The diagnosis of TMJ disease was performed on the presence of at least one Research Diagnostic Criteria for Temporomandibular Disorders (RDC/TMD) diagnosis. The anamnestic and functional data were collected in a medical record used by orthodontists of University of Pavia, Italy. 50,1% of the pts showed recurrent pain localized in the temporomandibular area, crepitation and jaw stiffness or fatigue. All TMJs werw examined by panoramic X-ray, teleradiography with latero-lateral and anteror-posterior view, and by SE by Esaote MyLAB 70 (Genoa Italy linear probe 8-13 Mhz).

## Results

40/40 (100%) O-JIA, 18/27 (66%) P-JIA and 2/9 (22%) ERA-JIA pts showed monolateral X-ray damage of the condyle and 4/27 (14,8%) P-JIA, 3 of 4 (75%) S-JIA and 1/9 (11,1%) ERA-JIA pts showed bilateral X-ray damage of the condyle. 34/40 (56,6%) O-JIA pts showed JE by SE and in 25 pts JE was bilateral 20/27 (74%) P-JIA pts showed JE and in 17 pts was bilateral. 2/4 (50%) S-JIA pts showed bilateral JE. In all pts SE showed bone remodeling of the condyle head, and in 30/40 (75%) O-JIA were present monolateral erosions. 40/40 (100%) O-JIA, 12/27 (44%) P-JIA pts showed clinically and teleradiography the facial asymmetry (p<0,005).

## Conclusions

Our data confirm that inflammation of TMJ in JIA pts may compromise the growth cartilage and rapidly progress into bone erosion of the condylar head leading to irreversible damage as micrognathia, aberrations in mandibulofacial development, and facial asymmetry. In this preliminary study, the results suggest early investigation of TMJ in all JIA pts, above all in O-JIA pts to avoid quickly and silent progression of bone damage. Most JIA children do not complain any symptoms and TMJ destruction remain unrecognized up to adolescence or early adulthood when the features of the disease may suddenly appear. An early treatment should prevent the irreversible and often intractable damage.

